# Examining the provisional guidelines for weight gain in twin pregnancies: a retrospective cohort study

**DOI:** 10.1186/s12884-017-1530-2

**Published:** 2017-09-29

**Authors:** Olha Lutsiv, Adam Hulman, Christy Woolcott, Joseph Beyene, Lucy Giglia, B. Anthony Armson, Linda Dodds, Binod Neupane, Sarah D. McDonald

**Affiliations:** 1 Department of Obstetrics and Gynecology, McMaster University, 1280 Main Street West, Room 3N52B, Hamilton, ON L8S 4K1 Canada; 2 Departments of Obstetrics and Gynaecology, and Pediatrics, Dalhousie University, Halifax, NS Canada; 3 Department of Clinical Epidemiology & Biostatistics, McMaster University, Hamilton, ON Canada; 4 Department of Pediatrics, McMaster University, Hamilton, ON Canada; 5 Department of Obstetrics and Gynaecology, Dalhousie University, Halifax, NS Canada; 6 Departments of Obstetrics and Gynecology, Radiology, and Clinical Epidemiology & Biostatistics, McMaster University, Hamilton, ON Canada

**Keywords:** Guidelines, Pregnancy, Small for gestational age, Twins, Weight gain

## Abstract

**Background:**

Weight gain during pregnancy has an important impact on maternal and neonatal health. Unlike the Institute of Medicine (IOM) recommendations for weight gain in singleton pregnancies, those for twin gestations are termed “provisional”, as they are based on limited data. The objectives of this study were to determine the neonatal and maternal outcomes associated with gaining weight below, within and above the IOM provisional guidelines on gestational weight gain in twin pregnancies, and additionally, to explore ranges of gestational weight gain among women who delivered twins at the recommended gestational age and birth weight, and those who did not.

**Methods:**

A retrospective cohort study of women who gave birth to twins at ≥20 weeks gestation, with a birth weight ≥ 500 g was conducted in Nova Scotia, Canada (2003–2014). Our primary outcome of interest was small for gestational age (<10th percentile). In order to account for gestational age at delivery, weekly rates of 2nd and 3rd trimester weight gain were used to categorize women as gaining below, within, or above guidelines. We performed traditional regression analyses for maternal outcomes, and to account for the correlated nature of the neonatal outcomes in twins, we used generalized estimating equations (GEE).

**Results:**

A total of 1482 twins and 741 mothers were included, of whom 27%, 43%, and 30% gained below, within, and above guidelines, respectively. The incidence of small for gestational age in these three groups was 30%, 21%, and 20%, respectively, and relative to gaining within guidelines, the adjusted odds ratios were 1.44 (95% CI 1.01–2.06) for gaining below and 0.92 (95% CI 0.62–1.36) for gaining above. The gestational weight gain in women who delivered twins at 37–42 weeks with average birth weight ≥ 2500 g and those who delivered twins outside of the recommend ranges were comparable to each other and the IOM recommendations.

**Conclusions:**

While gestational weight gain below guidelines for twins was associated with some adverse neonatal outcomes, additional research exploring alternate ranges of gestational weight gain in twin pregnancies is warranted, in order to optimize neonatal and maternal outcomes.

**Electronic supplementary material:**

The online version of this article (10.1186/s12884-017-1530-2) contains supplementary material, which is available to authorized users.

## Background

Weight gain during pregnancy is increasingly recognized as being a key, modifiable perinatal factor with an important impact on a number of maternal and infant outcomes [1]. Recognizing the importance of gestational weight gain (GWG), in 2009 the US Institute of Medicine (IOM) released a guideline specifying the recommended amounts of weight that women with singleton gestations should gain during their pregnancy, depending on their pre-pregnancy body mass index (BMI) [2]. Since the research on GWG in multiple gestations was limited, only “provisional” recommendations regarding the optimal GWG in twin pregnancies were released at the time.

Despite accounting for only 3% of births [3], disproportionately more twins than singletons experience morbidity and mortality, occupying 28% of neonatal intensive care unit (NICU) days [4–6]. Small for gestational age (SGA; defined as birth weight < 10th percentile according to singleton cut-offs) affects more twins (14–20% of twin births), as does low birth weight (LBW) [5]. Due to the morbidity and mortality associated with small infant size, the current IOM guidelines on GWG are designed to reduce the risk of small infant size as well as preterm birth (PTB). Since the incidence of twin pregnancies is likely to continue to increase due to delayed child bearing and assisted reproductive technologies [7], determining the optimal GWG range for this population is of great importance.

While some raise caution at the fact that excessive weight gain during pregnancy may be detrimental to the mother and her baby [8], others have speculated that the provisional GWG recommendations for twin gestations may not be high enough to prevent LBW [9]. GWG above the IOM guidelines for singleton pregnancies is associated with significant adverse maternal outcomes, including pre-eclampsia and overweight/obesity later in life [10–12], and neonatal outcomes, such as high birth weight, which in turn predisposes them to overweight/obesity in adolescence [8, 13, 14]. Therefore, the benefits of higher GWG for reducing small infant size in twins need to be offset against the possible adverse effects associated with excessive gain [15].

Although several studies have since attempted to explore the adequacy of the provisional IOM GWG guidelines for twins, no consensus has yet been reached. Due to this, more current, robust research to guide optimal GWG in twin pregnancies has been called for [16].

The objectives of this study were two-fold: 1) to examine the association between the existing provisional IOM GWG guidelines for twin pregnancies and SGA (and other secondary maternal and neonatal outcomes), and 2) to determine the GWG in women who delivered twins at the recommended gestational age and birth weight, using a large cohort separate from that in which the provisional guidelines were estimated, in order to explore similarities and/ or differences in optimal GWG.

## Methods

### Study design and data source

A retrospective cohort study was conducted of all women who gave birth to twins between January 1, 2003 and December 31, 2014 in Nova Scotia, which is an eastern province of Canada. Data for this study were obtained from the Nova Scotia Atlee Perinatal Database (NSAPD), a validated population–based database that captures information on all births within the province [17–19].

The NSAPD contains maternal and neonatal information on demographics, procedures/ interventions, diagnoses, morbidities and mortality for all pregnancies and births, which is extracted from antenatal and medical charts by trained personnel, using standardized forms. The NSAPD is a valid and reliable database, as confirmed by an ongoing quality assurance program, which carries out periodic abstraction studies.

### Inclusion and exclusion criteria

Women who were between 18 and 45 years of age and gave birth to twins at ≥20 weeks were eligible for study inclusion. Women were excluded if one or both of their infants had a birth weight < 500 g, major congenital anomalies, twin-to-twin transfusion syndrome, or if they were conjoined or monoamniotic. Additionally, mothers of single infants with a co-twin loss, mothers of co-twins of infants lost presumably from pregnancies that started with >2 fetuses, and mothers of infants with undetermined, unknown, or missing chorionicity were also excluded. Records with missing data on gestational age at delivery, BMI, or maternal weight at the time of delivery were excluded. Women who were underweight (BMI < 18.5 kg/m^2^) were excluded since the current IOM guidelines do not provide GWG recommendations for underweight women with twin pregnancies [2].

### Exposure, outcome and other variables of interest

The primary exposure was maternal GWG, categorized as below, within, or above the IOM provisional guidelines for twin pregnancies, according to the woman’s pre-pregnancy BMI group (normal weight BMI 18.5–24.9 kg/m^2^, overweight 25.0–29.9 kg/m^2^ or obese ≥30.0 kg/m^2^). Women’s total GWG was calculated by subtracting their self-reported pre-pregnancy weight, or if unavailable, their first measured weight, from their last measured weight closest to delivery. The measure of pre-pregnancy weight as reported in the NSAPD is based on the value that is written by the physician on the Nova Scotia Prenatal Record (field: “Pre-Pregnancy Weight”). The NSAPD does not record the specific source of this information, however it is known that the physician can use a pre-pregnancy weight as reported by the mother or a weight at the first prenatal visit (which for most women will occur in the first trimester; the NSAPD does not capture the date of this visit).

Gestational age at delivery was based on the last menstrual period or ultrasound. The majority of the women (84.5%) in the NSAPD have an early-pregnancy ultrasound. In women with both gestational age estimates, 87.2% of the last menstrual period-based estimates are within the ultrasound-based estimates by +/− 1 completed week. The total GWG recommendations for twin pregnancies for normal weight, overweight and obese women are: 16.8–24.5 kg, 14.1–22.7 kg and 11.4–19.1 kg, respectively. According to the IOM, the average cumulative weight gains for the first trimester (up to 13 weeks of gestation) in women who deliver twins at the recommended gestational age and birth weight (i.e., gestational ages 37–42 weeks and an average twin birth weight > 2500 g) are: 3.6 kg, 2.1 kg and 2.0 kg, respectively, for the three BMI categories. The optimal length of gestation was not defined by the IOM, although they considered a gestational age of 37–42 weeks in their recommendations of total GWG. Since that time practice has shifted to twin delivery during the earlier portion of that range in most instances [20, 21]. For this reason, we assumed that a term twin pregnancy is 38 weeks, and thus the recommended 2nd and 3rd trimester weekly rates of weight gain were calculated for each BMI group according to the formula: (IOM recommended total GWG – IOM average cumulative GWG up to 13 weeks)/ (38 weeks – 13 weeks). A similar formula was applied to estimate each woman’s actual 2nd and 3rd trimester weekly rate of GWG for her BMI group. The women’s actual 2nd and 3rd trimester weekly rates of GWG were compared with the calculated IOM recommended rates of gain for their BMI group, and accordingly, women were categorized as gaining below, within, or above the guidelines.

Our primary outcome was SGA, defined as birth weight < 10th percentile for gestational age and sex based on singleton growth curves [22]. Singleton growth curves currently provide the best predictors of adverse outcomes in twins, and are thus recommended by obstetrical associations as the preferred method for evaluating growth abnormalities in twins [23]. According to the National Institute for Health and Care Excellence, no evidence-based growth charts specific to twin and triplet pregnancies are available for use [20].

Secondary neonatal outcomes included: birth weight (continuous outcome), LBW <2500 g, SGA more strictly defined as birth weight < 5th percentile for gestational age and sex, large for gestational age (LGA) defined as birth weight > 90th percentile for gestational age and sex, Apgar score < 7 at 5 min, umbilical cord pH <7.10, respiratory distress, hypoglycaemia, NICU admission, and NICU length of stay.

Secondary maternal outcomes included: gestational age at delivery (continuous outcome), PTB <37 weeks (overall and broken down into spontaneous and indicated), labour induction, mode of delivery (unassisted birth, instrumental birth, and Caesarean section), postpartum haemorrhage, and length of postpartum hospital stay.

Important covariates included: maternal age, ethnicity, marital status, smoking status and drug use during pregnancy, pre-existing diabetes mellitus, pre-existing hypertension, other physical health problems, mental health problems, prenatal class attendance, chorionicity, and babies’ sex. In addition to a woman’s level of education, a neighborhood-level measure of socioeconomic status was also included – the Quintile of Adjusted Income Per Person Equivalent (QAIPPE), where 1 corresponds to the lowest and 5 corresponds to the highest income quintiles [24]. Obstetrical history was also included: parity and past history of gestational diabetes, gestational hypertension, LBW, and neonatal death.

### Statistical analyses

Baseline characteristics were compared across the three GWG groups – women who gained below, within, or above the IOM GWG guidelines. Continuous variables were compared with the analysis of variance (ANOVA) or Kruskal-Wallis test. Categorical data were compared with a χ^2^ or Fisher’s exact test. To address our primary objective, to determine the effects of gaining below, within, or above the IOM GWG guidelines on each of the individual outcomes, linear regression was used for continuous outcomes and logistic regression was used for binary outcomes, with generalized estimating equations (GEE) used for the neonatal outcomes in order to adjust for the correlation between twins in a set. In order to assess the independent effect of GWG on neonatal and maternal outcomes, multivariable analyses were performed, adjusting for baseline maternal and pregnancy characteristics that may confound the associations of interest. The multivariable analyses adjusted for relevant a priori determined variables, which included maternal age, maternal pre-pregnancy BMI, smoking, socioeconomic status, parity, and chorionicity, as well as any additional baseline characteristics significant with a *p*-value <0.2 in the univariate analyses. All effect estimates from the models using GEE for the neonatal outcomes and the regression models for the maternal outcomes were reported as mean differences (MD; for continuous outcomes) or odds ratios (OR; for binary outcomes), with their accompanying 95% confidence intervals (CI).

Additionally, in order to explore potential GWG effect measure modification by parity or chorionicity, the multivariable models for all maternal and neonatal outcomes were also reanalyzed with an additional interaction term between parity and GWG, and separately chorionicity and GWG. The Wald test was used to test the significance of the interaction terms.

In order to address our second objective and determine the GWG in women who delivered twins at the recommended gestational age and birth weight, we followed the calculations outlined in the IOM guidelines in our study population. The recommended gestational age was defined as birth at 37–42 weeks of gestation. The recommended birth weight was defined as an average twin birth weight ≥ 2500 g. The interquartile range (IQR: 25th and 75th percentiles of the 2nd and 3rd trimester weekly rates of GWG) was determined. To reflect more recent clinical practice, we repeated the calculations after replacing 42 with 38 ^6/7^ weeks as the upper limit of gestational age at birth, and further amending the definition of the recommended birth weight, such that both twins individually weighed ≥2500 g. We also determined ranges of GWG in women who delivered twins outside of the recommended gestational age and birth weight, based on both definitions for comparison.

Prior to study commencement, a sample size calculation was performed. Analyses were performed using SAS (version 9.1; SAS Institute Inc., Cary, NC). We followed the *Strengthening the Reporting of Observational Studies in Epidemiology* (STROBE) guideline [25]. Institutional review board approval was obtained from the Faculty of Health Sciences/ McMaster University Research Ethics Board (#12-140C), the Reproductive Care Program of Nova Scotia Data Access Committee and the IWK Health Centre Research Ethics Board (#1012023) prior to study commencement.

## Results

**Fig. 1:**
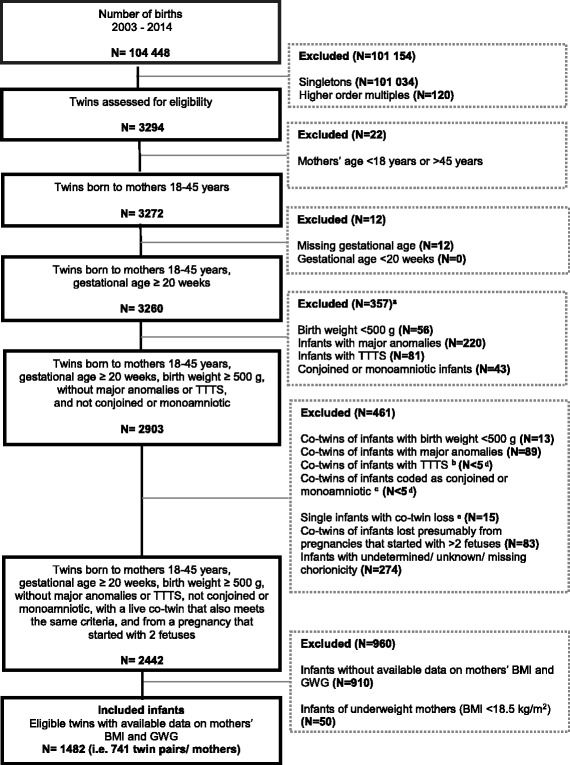
Flow Chart of Subject Selection. TTTS = Twin Twin Transfusion Syndrome. ^a^ some infants excluded for more than one reason. ^b^ the co-twin was excluded because they had been coded with TTTS, but the twin that remains in the database was not coded as having TTTS. ^c^ the co-twin was excluded for having twin type coded as monoamniotic, but the twin that remains in the database was not coded as having this twin type. ^d^ exact numbers cannot be reported in order to preserve confidentiality of the research subjects.^e^ of the infants without a co-twin in the dataset, these ones have a code corresponding to continuing pregnancy after spontaneous abortion of one fetus or more, after selective fetal reduction, or after intrauterine fetal demise – in other words, ICD10CA codes of O31.11×, O31.12×, or O31.2; there were no instances of O31.0 (papyraceous fetus)

There were 3294 twin hospital births to 1647 women between January 1, 2003 and December 31, 2014 in Nova Scotia (Fig. 1). After excluding records of women who did not meet our study criteria (*N* = 445), and those who had missing information on gestational age, maternal pre-pregnancy height or weight, or maternal weight at delivery (*N* = 461), 741 women and 1482 infants were included.

### Baseline characteristics

The majority of the women did not gain the IOM recommended amount of weight during pregnancy; 27.1% gained below, 29.7% gained above, and only 43.2% gained within the guidelines according to the 2nd and 3rd trimester weekly rates of weight gain. The median maternal age was 31 years (IQR 27, 34), with the majority of women being married or common-law (77.8%), overweight or obese according to their pre-pregnancy BMI classification (26.5% and 26.3%, respectively), multiparous (52.5%), and residing in the top two highest neighborhood-level income quintiles (4th and 5th quintile, 23.0% and 21.0%, respectively). Approximately one-fifth of the infants were monochorionic (19.4%), and 47.2% were male (with the sex of one baby being ambiguous). Additional baseline characteristics of all study participants are reported in Table 1, according to their IOM classification of GWG.

**Table 1:** Baseline characteristics of women with twin pregnancies according to gestational weight gain

*Baseline Characteristic*	All Participants (*N* = 741)	Below IOM ^a^GWG (*N* = 201)	Within IOM ^a^GWG (*N* = 320)	Above IOM ^a^GWG (*N* = 220)	
	*N* (%) ^b^	*N* (%) ^b^	*N* (%) ^b^	*N* (%) ^b^	*P* value ^c^
Maternal age, years, median (IQR)	31 (27, 34)	29 (25, 34)	32 (28, 35)	30 (26, 34)	<0.001
Caucasian	318 (82.8)	91 (82.0)	134 (84.3)	93 (81.6)	0.81
Married or common-law	556 (77.8)	145 (74.7)	255 (82.5)	156 (73.6)	0.03
Post-secondary education or higher	131 (50.4)	33 (47.1)	66 (56.4)	32 (43.8)	0.20
Neighborhood-level income quintile					0.36
1st quintile	139 (19.9)	46 (23.7)	51 (17.1)	42 (20.4)	
2nd quintile	109 (15.6)	33 (17.0)	46 (15.4)	30 (14.6)	
3rd quintile	143 (20.5)	38 (19.6)	57 (19.1)	48 (23.3)	
4th quintile	161 (23.0)	45 (23.2)	70 (23.4)	46 (22.3)	
5th quintile	147 (21.0)	32 (16.5)	75 (25.1)	40 (19.4)	
Pre-pregnancy BMI, kg/m^2^, median (IQR)	25.5 (22.5, 30.3)	26.0 (22.6, 32.7)	25.0 (22.1, 29.5)	25.7 (23.0, 30.1)	0.07
Pre-pregnancy BMI classification					0.15
Normal weight (BMI 18.5 to 24.9 kg/m^2^)	350 (47.2)	93 (46.3)	161 (50.3)	96 (43.6)	
Overweight (BMI 25.0 to 29.9 kg/m^2^)	196 (26.5)	45 (22.4)	84 (26.3)	67 (30.5)	
Obese (BMI ≥ 30.0 kg/m^2^)	195(26.3)	63 (31.3)	75 (23.4)	57 (25.9)	
Parity ≥1	390 (52.6)	119 (59.2)	172 (53.8)	99 (45.0)	0.01
Previous gestational diabetes ^d^	15 (2.0)	NA	NA	NA	0.76
Previous gestational hypertension	29 (3.9)	8 (4.0)	14 (4.4)	7 (3.2)	0.78
Previous Caesarean section	86 (11.7)	20 (10.1)	40 (12.6)	26 (12.0)	0.68
Previous LBW ^d^	20 (2.8)	NA	NA	NA	0.31
Previous neonatal death	7 (1.0)	NA	NA	NA	0.31
Smoking during pregnancy	68 (10.3)	30 (17.2)	23 (7.8)	15 (7.9)	0.002
Drug use during pregnancy ^d^	15 (2.0)	NA	NA	NA	0.64
Pre-existing diabetes mellitus ^d^	9 (1.2)	NA	NA	NA	0.002
Pre-existing hypertension ^d^	15 (2.0)	NA	NA	NA	0.32
Other physical health problems	194 (26.2)	57 (28.4)	84 (26.3)	53 (24.1)	0.61
Mental health problems	69 (9.3)	17 (8.5)	29 (9.1)	23 (10.5)	0.76
Attended prenatal classes	143 (41.9)	42 (43.3)	50 (35.2)	51 (50.0)	0.07
Twin type					0.52
Monochorionic – diamniotic	144 (19.4)	42 (20.9)	52 (16.3)	50 (22.7)	
Dichorionic (dissimilar sexes or blood groups)	294 (39.7)	77 (38.3)	128 (40.0)	89 (40.5)	
Dichorionic (similar sexes and blood groups)	135 (18.2)	34 (16.9)	64 (20.0)	37 (16.8)	
Dichorionic (similar sexes but blood groups undetermined)	168 (22.7)	48 (23.9)	76 (23.7)	44 (20.0)	
Sex of the baby ^e^					0.40
Male	699 (47.2)	203 (50.5)	294 (45.9)	202 (46.0)	
Female	782 (52.8)	199 (49.5)	346 (54.1)	237 (54.0)	

Abbreviations: BMI, body mass index; GWG, gestational weight gain; IOM, Institute of Medicine; IQR, inter-quartile range; N, number; NA, not available

^a^ GWG categories (below, within, and above IOM GWG) are based on the 2nd and 3rd trimester weekly rates of GWG

^b^ Baseline characteristics are mostly reported as N (%), unless otherwise specified (i.e., median (IQR))

^c^ *P* values were calculated with the Kruskal-Wallis test for continuous variables and with the χ^2^ test or Fisher’s exact test for categorical variables

^d^ The proportions of previous gestational diabetes, previous LBW, previous neonatal death, drug use during pregnancy, pre-existing diabetes mellitus, and pre-existing hypertension could not be reported by GWG category in order to preserve the confidentiality of the research subjects, since some cells had <5 events

^e^ Sex of the baby is reported at the neonatal level and not the maternal level, thus total N = 1482; the sex of one baby was ambiguous; the *p*-value is based on the analysis of generalized estimating equations parameter estimates

Women who were excluded from our analyses due to missing information (*N* = 461) had similar baseline characteristics as those included (Additional file 1: Table S1).

### Objective 1: Neonatal and maternal outcomes according to IOM classification of GWG

Our primary outcome, SGA <10th percentile, occurred in 30.1% of neonates exposed to inadequate maternal weight gain, which was significantly higher than in neonates exposed to appropriate (21.1%) or excess maternal weight gain (19.8%, Table 2). After controlling for potential confounders, neonates of women who gained below recommendations had a 44% higher odds of SGA <10th percentile (95% CI 1.01 to 2.06) than neonates of women who gained within the guidelines (Table 3). They also had higher odds of LBW (adjusted OR 1.55, 95% CI 1.07 to 2.23), and a longer NICU stay (mean + 4.4 days, 95% CI 0.7 to 8.2 days). The adjusted odds of SGA or any secondary neonatal outcome was not significantly different in women who gained above the guidelines compared to those who gained within. Women who gained weight above the provisional guidelines had higher odds of labour induction (adjusted OR 1.65 [95% CI 1.08, 2.53], Tables 4 and 5).

**Table 2:** Outcomes of twins according to mothers’ gestational weight gain

Neonatal Outcome	All Participants (*N* = 1482)	Below IOM ^a^GWG (*N* = 402)	Within IOM ^a^GWG (*N* = 640)	Above IOM ^a^GWG (*N* = 440)	
	N (%) ^b^	N (%) ^b^	N (%) ^b^	N (%) ^b^	P value ^c^
SGA <10th percentile	343 (23.2)	121 (30.1)	135 (21.1)	87 (19.8)	0.004
SGA <5th percentile	193 (13.0)	69 (17.2)	85 (13.3)	39 (8.9)	0.005
LBW <2500 g	603 (40.7)	204 (50.8)	244 (38.1)	155 (35.2)	<0.001
LGA >90th percentile	31 (2.1)	5 (1.2)	11 (1.7)	15 (3.4)	0.22
Birth weight, g, median (IQR)	2603 (2260, 2885)	2475 (2100, 2765)	2653 (2290, 2905)	2637 (2356, 2910)	<0.001
Apgar score < 7 at 5 min	39 (2.6)	13 (3.3)	10 (1.6)	16 (3.7)	0.16
Umbilical cord pH <7.10	33 (2.7)	10 (2.9)	15 (2.8)	8 (2.2)	0.84
Respiratory distress	148 (10.0)	46 (11.4)	53 (8.3)	49 (11.1)	0.27
Hypoglycemia	160 (10.8)	43 (10.7)	71 (11.1)	46 (10.5)	0.96
NICU admission	555 (37.5)	170 (42.3)	230 (35.9)	155 (35.2)	0.21
NICU length of stay, days, median (IQR)	11 (2, 20)	13 (2, 23)	10 (2, 19)	11 (1, 20)	0.10

Abbreviations: BMI, body mass index; GWG, gestational weight gain; IOM, Institute of Medicine; IQR, inter-quartile range; LBW, low birth weight; LGA, large for gestational age (and sex); N, number; NICU, neonatal intensive care unit; SGA, small for gestational age (and sex)

^a^ Gestational weight gain categories (below, within, and above IOM GWG) are based on the 2nd and 3rd trimester weekly rates of GWG

^b^ Outcomes are mostly reported as N (%), unless otherwise specified (i.e., median (IQR))

^c^ *P* values are based on the analysis of generalized estimating equations parameter estimates

**Table 3:** Unadjusted and adjusted associations between gestational weight gain and neonatal outcomes

	Below IOM GWG ^a^		Above IOM GWG ^a^	
Neonatal Outcome	Unadjusted Analyses	Adjusted Analyses ^b^	Unadjusted Analyses	Adjusted Analyses ^b^
	OR (95% CI)	OR (95% CI)	OR (95% CI)	OR (95% CI)
SGA <10th percentile	1.61 (1.18, 2.21)	1.44 (1.01, 2.06)	0.92 (0.66, 1.29)	0.92 (0.62, 1.36)
SGA <5th percentile	1.35 (0.93, 1.98)	1.07 (0.69, 1.65)	0.64 (0.40, 1.00)	0.67 (0.39, 1.14)
LBW <2500 g	1.67 (1.23, 2.28)	1.55 (1.07, 2.23)	0.88 (0.65, 1.20)	0.81 (0.58, 1.14)
Birth weight, g ^c^	−1523 (−235, −71)	−145 (−233, −57)	18 (−64, 100)	39 (−48, 126)
Respiratory distress	1.43 (0.87, 2.36)	1.14 (0.65, 2.01)	1.39 (0.85, 2.27)	1.09 (0.63, 1.87)
Hypoglycemia	0.96 (0.60, 1.53)	0.73 (0.41, 1.28)	0.94 (0.60, 1.46)	1.13 (0.69, 1.84)
NICU admission	1.31 (0.94, 1.82)	1.29 (0.87, 1.91)	0.97 (0.70, 1.35)	0.92 (0.63, 1.35)
NICU length of stay, days ^c^	3.76 (0.32, 7.21)	4.45 (0.69, 8.20)	0.92 (−2.38, 4.22)	1.59 (−2.04, 5.22)

Abbreviations: BMI, body mass index; CI; confidence interval; GWG, gestational weight gain; IOM, Institute of Medicine; IQR, inter-quartile range; LBW, low birth weight; N, number; NICU, neonatal intensive care unit; OR, odds ratio; PTB, preterm birth; SGA, small for gestational age (and sex)

^a^ Gestational weight gain categories (below, within, and above IOM GWG) are based on the 2nd and 3rd trimester weekly rates of GWG; GWG within the IOM GWG is the referent group for all analyses

^b^ All outcomes were adjusted for the a priori defined confounders, including maternal age, neighborhood-level income, maternal pre-pregnancy BMI, parity, smoking status, and chorionicity. Analyses were also adjusted for the baseline characteristics significant with a *p-value* < 0.2, including marital status. Despite having a *p*-value <0.2, pre-existing diabetes mellitus and attending prenatal classes were not included in the adjusted models, due to low frequency of occurrence in the three categories of GWG (pre-existing diabetes mellitus), or collinearity with other variables in the model (attending prenatal classes)

^c^ The corresponding effect estimates are a mean difference (95% CI), instead of OR (95% CI)

**Table 4:** Outcomes of women with twin pregnancies by gestational weight gain

Maternal Outcome	All Participants (N = 741)	Below IOM ^a^GWG (*N* = 201)	Within IOM ^a^GWG (*N* = 320)	Above IOM ^a^GWG (*N* = 220)	
	*N* (%) ^b^	*N* (%) ^b^	*N* (%) ^b^	*N* (%) ^b^	*P* value ^c^
Gestational age at delivery, weeks, median (IQR)	37.0 (35.7, 38.0)	36.9 (35.6, 38.0)	37.1 (35.7, 38.0)	37.0 (35.7, 37.9)	0.49
PTB <37 weeks					
Overall	353 (47.6)	104 (51.7)	142 (44.4)	107 (48.6)	0.25
Spontaneous	155 (20.9)	49 (24.4)	67 (20.9)	39 (17.7)	0.25
Indicated	198 (26.7)	55 (27.4)	75 (23.4)	68 (30.9)	0.15
Labour induction	235 (31.7)	67 (33.3)	86 (26.9)	82 (37.3)	0.03
Mode of delivery					
Vaginal birth (unassisted)	304 (41.0)	98 (48.8)	125 (39.1)	81 (36.8)	0.03
Forceps/ vacuum	50 (6.8)	12 (6.0)	25 (7.8)	13 (5.9)	0.60
Caesarean section	387 (52.2)	91 (45.3)	170 (53.1)	126 (57.3)	0.04
Postpartum haemorrhage	148 (20.0)	42 (20.9)	63 (19.7)	43 (19.6)	0.93
Length of stay, days, median (IQR)	3.4 (2.7, 4.3)	3.2 (2.5, 4.1)	3.5 (2.8, 4.3)	3.6 (2.8, 4.6)	0.02

Abbreviations: BMI, body mass index; GWG, gestational weight gain; IOM, Institute of Medicine; IQR, inter-quartile range; N, number; PTB, preterm birth

^a^ Gestational weight gain categories (below, within, and above IOM GWG) are based on the 2nd and 3rd trimester weekly rates of GWG

^b^ Outcomes are mostly reported as N (%), unless otherwise specified (i.e., median (IQR))

^c^ *P* values were calculated with the Kruskal-Wallis test for continuous variables and with the χ^2^ test or Fisher’s exact test for categorical variables

**Table 5:** Unadjusted and adjusted associations between gestational weight gain and maternal outcomes

	Below IOM GWG ^a^		Above IOM GWG ^a^	
Maternal Outcome	Unadjusted Analyses	Adjusted Analyses ^b^	Unadjusted Analyses	Adjusted Analyses ^b^
	OR (95% CI)	OR (95% CI)	OR (95% CI)	OR (95% CI)
Gestational age at delivery, weeks ^c^	−0.28 (−0.66, 0.10)	−0.34 (−0.76, 0.07)	−0.10 (−0.47, 0.27)	0.01 (−0.39, 0.41)
PTB <37 weeks				
Overall	1.34 (0.94, 1.91)	1.28 (0.86, 1.93)	1.19 (0.84, 1.68)	1.09 (0.73, 1.62)
Spontaneous	1.22 (0.80, 1.85)	1.16 (0.71, 1.90)	0.81 (0.53, 1.26)	0.78 (0.47, 1.30)
Indicated	1.23 (0.82, 1.84)	1.20 (0.76, 1.91)	1.46 (0.99, 2.15)	1.34 (0.86, 2.09)
Labour induction	1.36 (0.93, 2.00)	1.54 (0.99, 2.39)	1.62 (1.12, 2.34)	1.65 (1.08, 2.53)
Mode of delivery				
Vaginal birth (unassisted)	1.48 (1.04, 2.12)	1.47 (0.96, 2.25)	0.91 (0.64, 1.30)	1.03 (0.67, 1.56)
Forceps/ vacuum	0.75 (0.37, 1.53)	0.91 (0.40, 2.09)	0.74 (0.37, 1.48)	0.73 (0.32, 1.67)
Caesarean section	0.73 (0.51, 1.04)	0.71 (0.46, 1.08)	1.18 (0.84, 1.67)	1.07 (0.71, 1.61)
Postpartum haemorrhage	1.08 (0.70, 1.67)	1.10 (0.67, 1.81)	0.99 (0.64, 1.53)	1.01 (0.62, 1.63)
Length of stay, days ^c^	−0.30 (−0.60, −0.01)	−0.35 (−0.68, −0.02)	0.03 (−0.25, 0.32)	−0.04 (−0.36, 0.28)

Abbreviations: BMI, body mass index; CI; confidence interval; GWG, gestational weight gain; IOM, Institute of Medicine; IQR, inter-quartile range; N, number; OR, odds ratio; PTB, preterm birth

^a^ Gestational weight gain categories (below, within, and above IOM GWG) are based on the 2nd and 3rd trimester weekly rates of GWG; GWG within the IOM GWG is the referent group for all analyses

^b^ All outcomes were adjusted for the a priori defined confounders, including maternal age, neighborhood-level income, maternal pre-pregnancy BMI, parity, smoking status, and chorionicity. Analyses were also adjusted for the baseline characteristics significant with a *p-value* < 0.2, including marital status. Despite having a p-value <0.2, pre-existing diabetes mellitus and attending prenatal classes were not included in the adjusted models, due to low frequency of occurrence in the three categories of GWG (pre-existing diabetes mellitus), or collinearity with other variables in the model (attending prenatal classes)

^c^ The corresponding effect estimates are a mean difference (95% CI), instead of OR (95% CI)

Gestational age, PTB <37 weeks, instrumental delivery, or postpartum hemorrhage did not differ by groups of GWG. The low incidence of LGA >90th percentile, Apgar score < 7 at 5 min, and umbilical cord pH <7.10, prohibited their exploration with multivariable regression. We did not find evidence of interaction between the classification of GWG and either parity or chorionicity (i.e., all *p* > 0.05 for interaction terms).

### Objective 2: GWG in women who delivered twins within and outside the recommended gestational age and birth weight

**Fig. 2:**
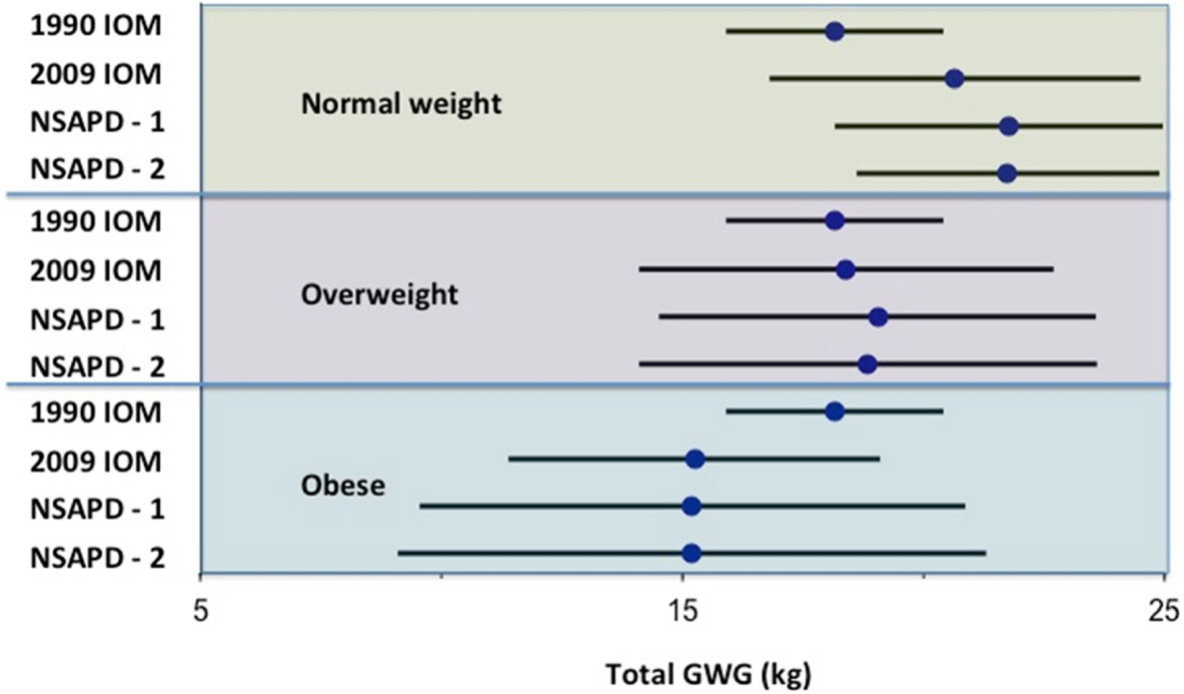
Total GWG in women with optimal outcomes in NSAPD in comparison to IOM provisional recommendations^a^. Circle represents the median GWG and the whiskers represent the 25th and 75th percentiles of GWG. Abbreviations: GWG, gestational weight gain; IOM, Institute of Medicine; NSAPD, Nova Scotia Atlee Perinatal Database. a Optimal outcome for NSAPD - 1 defined as birth at 37–42 weeks of gestation and average twin birth weight ≥ 2500 g; Optimal outcome for NSAPD - 2 defined as birth at 37–38 6/7 weeks of gestation, with both twins individually having a birth weight ≥ 2500 g

Forty-four percent of the women in our study population delivered twins between 37 and 42 weeks, with an *average* twin birth weight ≥ 2500 g, while 31% of the women delivered twins between 37 and 38 ^6/7^weeks, with *each* twin individually having a birth weight ≥ 2500 g. The rates of 2nd and 3rd trimester weight gain appeared to be fairly comparable between women who delivered within the recommend gestational age and birth weight ranges and those who did not, regardless of which definition was used, and were also similar to the provisional recommendations from the IOM, regardless of pre-pregnancy BMI (Table 6). Examining the total GWG in women who delivered at the recommended gestational age and birth weight reveals a trend towards higher optimal GWG in normal weight and overweight women compared to the IOM recommendations, but a lower optimal GWG in obese women (Fig. 2).

**Table 6:** The 2nd-3rd trimester weekly GWG in women with twin pregnancies with optimal and suboptimal outcomes

	Normal weight BMI 18.5–24.9 kg/m^2^	Overweight BMI 25.0–29.9 kg/m^2^	Obese BMI ≥ 30 kg/m^2^
	25th – 75th percentiles of 2nd + 3rd trimester weekly GWG (kg/week)^a^		
IOM provisional guideline	0.53–0.84	0.48–0.82	0.38–0.68
IOM definition in NSAPD			
Optimal outcome ^b^	0.58–0.87	0.49–0.85	0.30–0.77
Sub-optimal outcome	0.45–0.85	0.52–0.87	0.34–0.72
New definition in NSAPD			
Optimal outcome ^c^	0.61–0.86	0.48–0.88	0.29–0.80
Sub-optimal outcome	0.45–0.85	0.52–0.86	0.33–0.72

Abbreviations: BMI, body mass index; GWG, gestational weight gain; IOM, Institute of Medicine; NSAPD, Nova Scotia Atlee Perinatal Database

^a^ 2nd and 3rd trimester weekly GWG were calculated according to the formula: (Total GWG – IOM Average Cumulative GWG up to 13 weeks) / (Gestational Age – 13). The IOM Average Cumulative GWGs up to 13 weeks were 3.6 kg, 2.1 kg and 2.0 kg in normal weight, overweight and obese women

^b^ Optimal outcome defined as birth at 37–42 weeks and average twin birth weight ≥ 2500 g

^c^ Optimal outcome defined as birth at 37–38 ^6/7^ weeks and birth weight of both twins individually ≥2500 g

## Discussion

In this large retrospective cohort study, we determined that relative to gaining within the provisional IOM guidelines for twins, gaining above the guidelines was associated with higher odds of labour induction, while gaining below was associated with higher odds of SGA, LBW, and longer NICU stay. Additionally, we determined that the rates of 2nd and 3rd trimester weight gain for women who delivered twins *within* the recommended gestational age and birth weight ranges, of all pre-pregnancy BMI classes, were fairly comparable to the rates computed based on current recommendations by the IOM, but they were not very different from the rates in women who delivered *outside* of the recommended gestational age and birth weight ranges.

The original 1990 IOM guidelines recommended 6–20 kg of GWG for women carrying twins, regardless of their BMI [26]. Given the concerns of small infant size that are associated with low GWG, the IOM revised their guidelines in 2009 to recommend significantly higher GWG (17–25 kg for normal weight women, 14–23 kg for overweight women, and 11–19 kg for obese women) [2]. These guidelines were “provisional”, a seemingly reasonable term given that they were based on: a single study; historical in nature (1979 to 1999); with potential for selection bias (four teaching hospitals); and inclusion of women who delivered between 37 and 42 weeks of gestation, despite the fact that delivery of twins is now recommended between 38 and 39 weeks, to reduce mortality and morbidity [27]; and a focus on twins *averaging* ≥ 2500 g, thereby precluding the ability to truly examine the association between GWG and individual small infant size [28]. The guidelines did not report on GWG among women with “suboptimal” outcomes (i.e., women who deliver outside of the recommended gestational age and birth weight), even though it is also important to consider the difference in GWG between women with “optimal” and “suboptimal” outcomes.

Several studies have attempted to examine the 2009 recommendations for twin gestations, with conflicting results. Only a few studies considered SGA <10th percentile specifically, and of those that did, one did not find a significant difference between women [29], while another found a lower incidence in normal weight women who gained within or above guidelines, but no differences in overweight or obese women [9]. A number of studies called into question the provisional recommendations as they found that weight gain in accordance with or in excess of the guidelines was associated with larger birth weight and decreased incidence of prematurity [30–32]. Other studies corroborated these results, as excessive GWG was associated with a larger birth weight, without any significant increases in other adverse pregnancy outcomes [33]. Conversely, another study found both inadequate and excess weight gain were associated with lower birth weight and prematurity [29].

Strengths of this study include the methodology used to classify GWG as below, within, or above guidelines which took into account the length of gestation as recommended by the IOM. Unlike numerous previous studies that restricted their study population to 37 weeks or more, excluding up to 50% of the population of interest [30, 34] and making it impossible to examine “optimal” and “suboptimal” outcomes, or those that assumed a uniform rate of weight gain throughout the pregnancy, our classifications were based on the estimated weekly rates of weight gain for the 2nd and 3rd trimesters, reflective of the slower trajectory of weight gain during the first trimester. This allowed us not only to include a larger sample of women, but much more importantly, did not eliminate women with adverse outcomes, such as PTB. Using a weekly rate of GWG further overcomes some of the bias that can affect results based on total GWG, given its inherent correlation with gestational age. It should be noted, however, that this method may not fully remove the effects of gestational length, as it assumes a certain amount of weight gain in the first trimester; if this assumption is incorrect for some women, it will misclassify them as having inadequate, adequate or excessive weight gain as defined by the IOM provisional guidelines [35, 36].

We maintained three BMI groups, rather than grouping together women gaining within and above the guidelines to compare them to women gaining below, which dismisses differences in the incidence of adverse outcomes between the first two groups, and could therefore result in biased effect estimates [30–32]. The large sample size is another notable strength, as it ensured adequate statistical power to control for a number of key confounding variables. Finally, a strength of this study is the use of GEE in order to account for correlation between twins, resulting in robust and valid effect estimates and CIs. Thus we overcame limitations of the majority of previous studies which analyzed all outcomes at the maternal level (instead of the individual neonate level) or which did not account for correlation between twins altogether [29, 31, 33], which could result in seemingly significant differences between groups even when there are none.

Limitations of this study include some missing data as sometimes occurs with large databases, for some variables of interest, including GWG. However, a comparison of the baseline characteristics of the women who were included in the study and those who were excluded due to missing information did not reveal any significant differences. Furthermore, missing data does not necessarily bias our associations, although our study findings should not be over-generalized to be representative of all pregnant women. Additionally, while our sample size was large enough for us to control for a number of key confounding variables, we were unable to perform analyses stratified by pre-pregnancy BMI, as such analyses would have been underpowered.

Limitations of the method used to develop the provisional guidelines [2] should also be noted. First of all, a comparison of GWG between the women who deliver twins within and outside of the recommended gestational age and birth weight, is an important one, however it is lacking in the IOM guidelines. The smaller the difference between these two groups, the less robust the recommendation. Furthermore, the width of the IOM recommended range of GWG was based on data from a arbitrarily chosen percentiles (25th and 75th) from data from a single study as noted above [28] and not on outcomes. In addition to gestational age and birth weight, other maternal and neonatal outcomes should have also been considered when determining the recommended ranges. Since the currently published studies have mostly aimed to evaluate the provisional guidelines, more attention should be focused on developing new methodologies for defining recommendations, determining whether alternate ranges of GWG correspond with better outcomes, and examining optimal and suboptimal outcomes according to GWG. This is especially critical at this time because if the provisional recommendations are interpreted to be the gold standard, then it will be more difficult to prove that an alternate recommendation may lead to better outcomes, and to change practice.

Future research is required to examine different patterns of GWG throughout pregnancy on maternal and neonatal outcomes, using a longitudinal approach with serial antenatal weight measurements. Determining the optimal GWG for underweight women, and further refining and narrowing the recommended ranges of GWG for normal weight, overweight and obese women is also key. Studying the effects of GWG on SGA defined according to twin growth charts will also be important, if standardized, validated twin growth charts that are based on rigorous data are developed and recommended in clinical practice guidelines.

## Conclusions

In summary, while GWG below the provisional guidelines for twins was associated with SGA and other adverse neonatal outcomes, GWG above the guidelines did not reduce the odds of SGA, and was further associated with adverse maternal outcomes, such as labour induction. As such, GWG recommendations outside of the provisional IOM guidelines may not be advisable, and further research is required to confirm the robustness of the provisional guidelines.

## Additional file

Additional file 1: Table S1. Baseline characteristics of women with twin pregnancies included in analyses and excluded due to missing information. (DOCX 21 kb)

## Abbreviations

ANOVA: Analysis of Variance
BMI: Body Mass Index
CI: Confidence Interval
GEE: Generalized Estimating Equations
GWG: Gestational Weight Gain
IOM: Institute of Medicine
IQR: Inter-Quartile Range
LBW: Low Birth Weight
LGA: Large for Gestational Age
MD: Mean Difference
NICU: Neonatal Intensive Care Unit
NSAPD: Nova Scotia Atlee Perinatal Database
OR: Odds Ratio
PTB: Preterm Birth
QAIPPE: Quintile of Adjusted Income Per Person Equivalent
SGA: Small for Gestational Age
STROBE: Strengthening the Reporting of Observational Studies in Epidemiology
