# Supplementary material for: Examining the provisional guidelines for weight gain in twin pregnancies: a retrospective cohort study
Source: BMC Pregnancy Childbirth. 2017 Sep 29;17:330. doi: 10.1186/s12884-017-1530-2 (PMC5622523; doi:10.1186/s12884-017-1530-2)
Supplement: Additional file 1: Table S1. — Baseline characteristics of women with twin pregnancies included in analyses and excluded due to missing information. (DOCX 21 kb) [file 12884_2017_1530_MOESM1_ESM.docx]

**Table S1.** Baseline characteristics of women with twin pregnancies included in analyses and excluded due to missing information

| **Variable** | **All Included Participants**  (N = 741) | **Women Excluded Due to Missing Information** (N = 461) |  |
| --- | --- | --- | --- |
|  | ***N* (%)** **^a^** | ***N* (%)** **^a^** | ***P* value ^b^** |
| **Maternal age**, years, median (IQR) | 31 (27, 34) | 31 (27, 35) | 0.75 |
| **Neighborhood-level income quintile** 1^st^ quintile 2^nd^ quintile 3^rd^ quintile 4^th^ quintile 5^th^ quintile | 139 (19.9) 109 (15.6) 143 (20.5) 161 (23.0) 147 (21.0) | 76 (17.6) 76 (17.6) 77 (17.8) 108 (25.0) 95 (22.0) | 0.58 |
| **Pre-pregnancy BMI classification** Normal weight (BMI 18.5 to 24.9 kg/m^2^) Overweight (BMI 25.0 to 29.9 kg/m^2^) Obese (BMI ≥ 30.0 kg/m^2^) | 350 (47.2) 196 (26.5) 195(26.3) | 68 (54.0) 26 (20.6) 32 (25.4) | 0.29 |
| **Parity ≥ 1** | 390 (52.6) | 256 (56.8) | 0.17 |
| **Smoking during pregnancy** | 68 (10.3) | 39 (10.5) | 0.91 |
|  |  |  |  |
| Abbreviations: BMI, body mass index; IQR, inter-quartile range; N, number  ^a^ Baseline characteristics are mostly reported as N (%), unless otherwise specified (i.e., median (IQR)).  ^b^ *P* values were calculated with the Kruskal-Wallis test for the continuous variable and with the χ^2^ test for categorical variables. | | | |
